# Supplementary material for: Serological Responses to Human Virome Define Clinical Outcomes of Italian Patients Infected with SARS-CoV-2
Source: medRxiv. 2020 Sep 7:2020.09.04.20187088. Preprint. [Version 1] doi: 10.1101/2020.09.04.20187088 (PMC7480049; doi:10.1101/2020.09.04.20187088)
Supplement: 1 — Supplementary Figure S1. Prevalence of viruses. (A) The number of unique epitopes and the composition of prevalence in cases with age ≥ 60 yrs (the median of all COVID cases) and age < 60 yrs. (B) The number of unique epitopes and the composition of prevalence in male and female cases. (C) The total reactivity of all epitopes in cases with age ≥ 60 yrs and age < 60 yrs. (D) The total reactivity of all epitopes in male and female cases. In violin plots, boxes span the interquartile range; lines within boxes represent the median; the width of violin plots indicates the kernel density of values. Supplementary Figure S2. Prevalence of serological responses to SARS-CoV virus in hospitalized patients with ICU (A-B) or death status (C). The width of violin plots indicates the kernel density of values. Supplementary Figure S3. Longitudinal progression of the normalized EBS across individuals. (A) Individual trajectories over time for patients grouped by hospital ward (gray lines), which were averaged (solid blue line) and fitted by linear regression (dashed blue line; slope and standard error shown in the legend). Baseline refers to the first sample obtained after admission to the hospital. Left: non-ICU; right: ICU. (B) Analogous results for patients grouped by outcome. Left: alive; right: deceased. Supplementary Figure S4. Sex and age effects of the humoral immune response of COVID-19 patients. (A-B) Total epitope enrichment at baseline as a function of age for male (A) and female (B) patients, respectively. (C-F) Longitudinal progression of the normalized EBS for younger males (C), older males (D), younger females (E), and older females (F), respectively. In violin plots, boxes span the interquartile range; lines within boxes represent the median; the width of violin plots indicates the kernel density of values. [file NIHPP2020.09.04.20187088-supplement-1.pdf]

medRxiv preprint doi: https://doi.org/10.1101/2020.09.04.20187088.this version posted September 7, 2020. The copyright holder for this preprint (which was not certified by peer review) is the author/funder, who has granted medRxiv a license to display the preprint in perpetuity.
This article is a US Government work. It is not subject to copyright under 17 USC 105 and is also made available for use under a CC0 license.

Suppl Figure S1

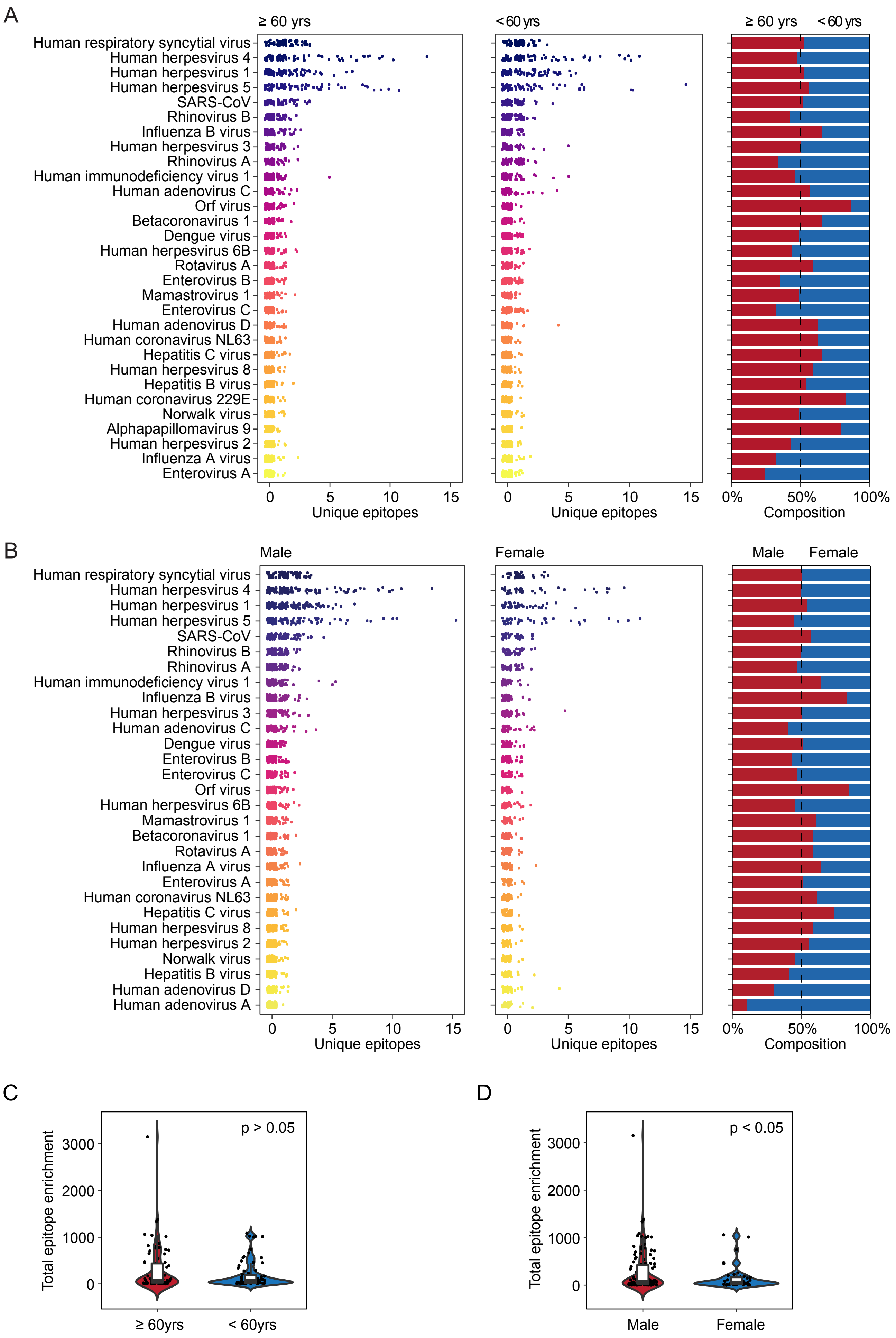

medRxiv preprint doi: https://doi.org/10.1101/2020.09.04.20187088.this version posted September 7, 2020. The copyright holder for this preprint
(which was not certified by peer review) is the author/funder, who has granted medRxiv a license to display the preprint in perpetuity.
This article is a US Government work. It is not subject to copyright under 17 USC 105 and is also made available
for use under a CC0 license.

Suppl Figure S2

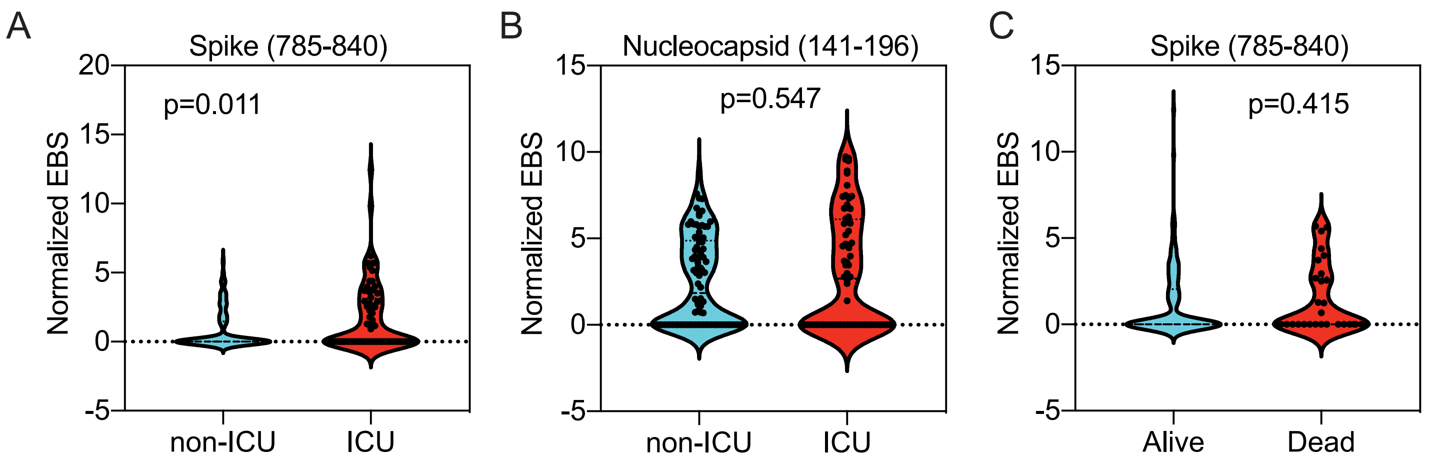

medRxiv preprint doi: https://doi.org/10.1101/2020.09.04.20187088.this version posted September 7, 2020. The copyright holder for this preprint
(which was not certified by peer review) is the author/funder, who has granted medRxiv a license to display the preprint in perpetuity.
This article is a US Government work. It is not subject to copyright under 17 USC 105 and is also made available
for use under a CC0 license.

Suppl Figure S3

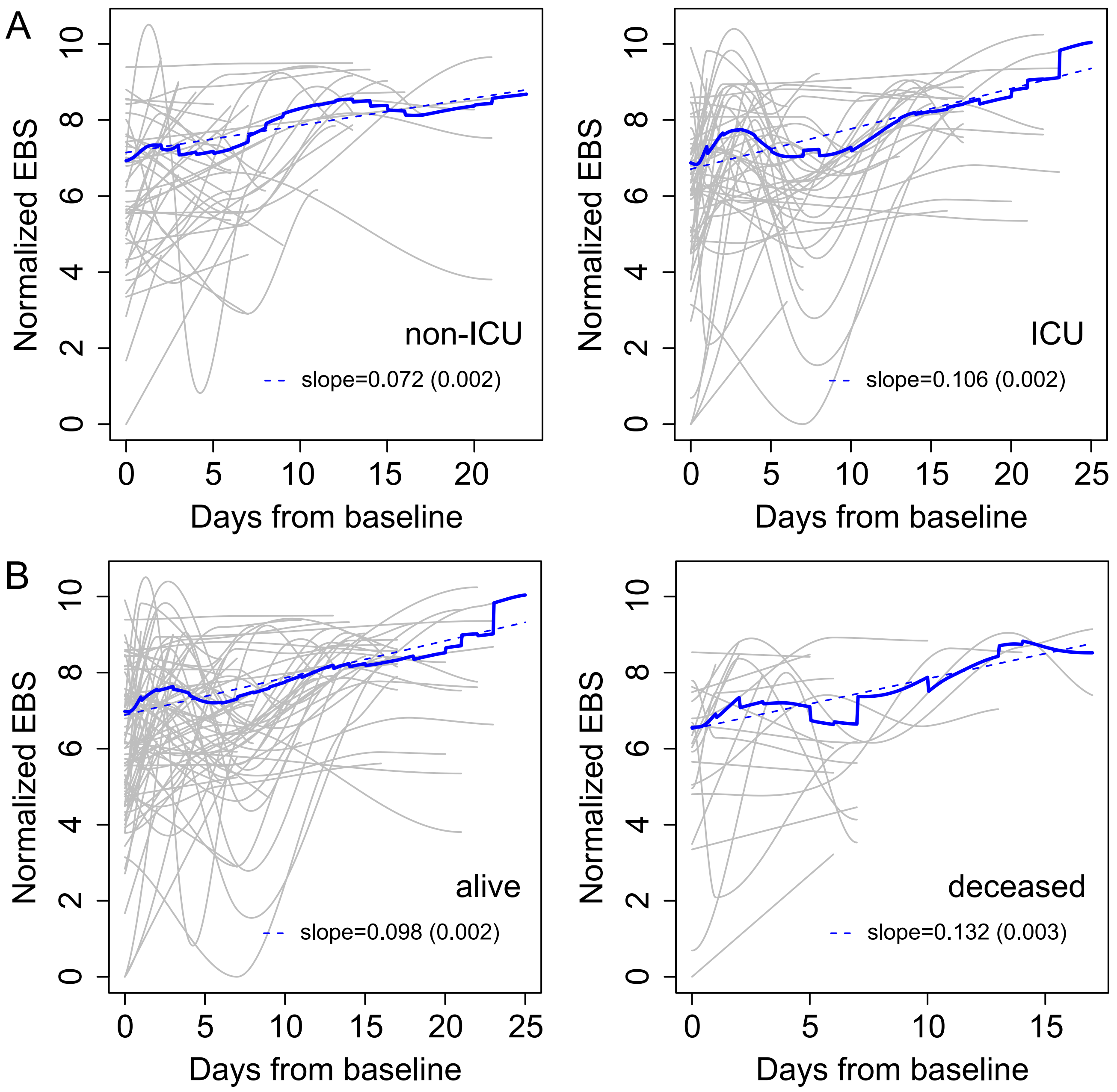

medRxiv preprint doi: https://doi.org/10.1101/2020.09.04.20187088.this version posted September 7, 2020. The copyright holder for this preprint (which was not certified by peer review) is the author/funder, who has granted medRxiv a license to display the preprint in perpetuity.
This article is a US Government work. It is not subject to copyright under 17 USC 105 and is also made available for use under a CC0 license.

Suppl Figure S4

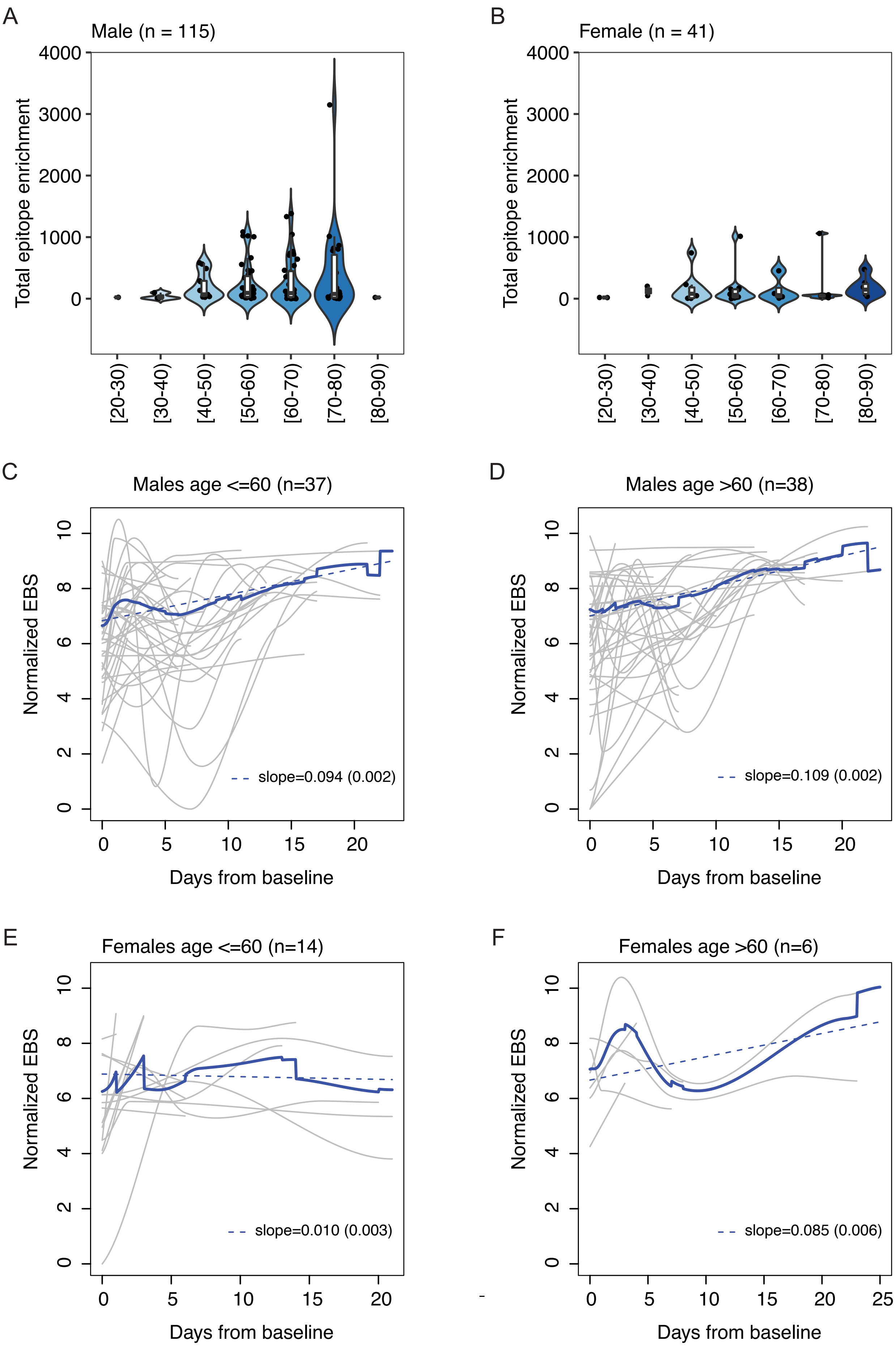

medRxiv preprint doi: https://doi.org/10.1101/2020.09.04.20187088.this version posted September 7, 2020. The copyright holder for this preprint (which was not certified by peer review) is the author/funder, who has granted medRxiv a license to display the preprint in perpetuity.
This article is a US Government work. It is not subject to copyright under 17 USC 105 and is also made available for use under a CC0 license.

Supplementary table 1. Viral prevalence in Brescia cohort. Related to Figure 1.

Virus species	Total (n=156)	Moderate (n=11)	Severe (n=19)	Critical (n=126)
Human respiratory syncytial virus	81%	64%	79%	83%
Human herpesvirus 4	74%	91%	95%	70%
Human herpesvirus 1	67%	55%	79%	66%
Human herpesvirus 5	55%	55%	63%	54%
SARS-CoV	48%	27%	53%	49%
Rhinovirus B	37%	27%	58%	34%
Rhinovirus A	33%	27%	47%	32%
Human immunodeficiency virus 1	23%	27%	32%	21%
Human herpesvirus 3	22%	9%	32%	22%
Influenza B virus	19%	9%	16%	21%
Human adenovirus C	17%	9%	21%	17%
Enterovirus B	14%	0%	16%	15%
Enterovirus C	13%	0%	16%	14%
Dengue virus	13%	9%	21%	12%
Human herpesvirus 6B	13%	9%	11%	13%
Mamastrovirus 1	10%	9%	11%	10%
Orf virus	10%	0%	16%	10%
Betacoronavirus 1	10%	0%	11%	10%
Rotavirus A	10%	0%	11%	10%
Enterovirus A	8%	0%	16%	7%
Influenza A virus	8%	9%	0%	9%
Human adenovirus D	7%	18%	0%	7%
Human coronavirus NL63	7%	0%	11%	7%
Human herpesvirus 8	6%	9%	0%	7%
Norwalk virus	6%	0%	21%	5%
Hepatitis B virus	6%	0%	11%	6%
Hepatitis C virus	6%	0%	0%	7%
Human herpesvirus 2	6%	9%	0%	6%
Human coronavirus 229E	4%	0%	5%	4%
Alphapapillomavirus 9	3%	0%	5%	3%
